# Whole genome sequence of an edible mushroom *Strobilomyces alpinus* (Boletaceae)

**DOI:** 10.1093/g3journal/jkaf080

**Published:** 2025-04-10

**Authors:** Li-Hong Han, Yan-Ting Duan, Han-Yan Yuan, Yan-Jia Hao, Chao Liu

**Affiliations:** College of Biological Resource and Food Engineering, Yunnan Engineering Research Center of Fruit Wine, Qujing Normal University, Qujing, 655011, China; College of Biological Resource and Food Engineering, Yunnan Engineering Research Center of Fruit Wine, Qujing Normal University, Qujing, 655011, China; College of Biological Resource and Food Engineering, Yunnan Engineering Research Center of Fruit Wine, Qujing Normal University, Qujing, 655011, China; School of Horticulture, Anhui Agricultural University, Hefei, 230036, China; College of Biological Resource and Food Engineering, Yunnan Engineering Research Center of Fruit Wine, Qujing Normal University, Qujing, 655011, China

**Keywords:** genome, macrofungi, *Strobilomyces alpinus*, secondary metabolism, genome assembly

## Abstract

*Strobilomyces alpinus* is a unique and significant mushroom endemic to southwestern and central China, characterized by its exclusive subalpine distribution and a strong host preference for *Abies* spp. The biological and genetic studies of this mushroom are scarce, which significantly hinders research on molecular breeding and evolutionary patterns. In this study, we report the de novo sequencing and assembly of the *S. alpinus* genome using the DNBSEQ-T7 and the third-generation Pacific Biosciences sequencing platform. The total genome size was approximately 58.75 Mb, with a GC content of 54.87%. The genome assembly produced 68 contigs, with an N50 length of 4.03 Mb. The genome comprises 11,761 annotated protein-coding genes, including 813 CAZyme-coding genes, 182 Cytochrome P450 genes, and 1,821 candidate pathogenicity-related genes. The non-coding RNA prediction results indicated the presence of 532 rRNAs, 62 small RNAs, and 98 tRNAs in the *S. alpinus* genome. Notably, there is a high degree of repetition (44.28%) within the *S. alpinus* genome. Additionally, we identified 16 secondary metabolite gene clusters, including 7 NRPS-like clusters, 5 terpene clusters, 1 fungal-RiPP-like cluster, 1 RiPP-like cluster, 1 T1PKS cluster, and 1 T1PKS-NRPS linkage gene cluster. Several important metabolic pathways, including terpenoid backbone biosynthesis, porphyrin metabolism, and folate biosynthesis, have been elucidated. The annotated whole-genome sequence of *S. alpinus* can serve as a reference for investigations of bioactive compounds with medicinal value and for commercial production.

## Introduction

The genus *Strobilomyces* Berk. (Boletaceae) is known as the “old man of the woods.” In the previous classification system (McNabb 1967), *Strobilomyces* was once categorized under the Strobilomycetaceae. However in 2001, it was reclassified into the Boletaceae (www.indexfungorum.org). Species within the genus *Strobilomyces* are globally distributed, with occurrences in Asia, Africa, Oceania, Europe, North America, and Central America. These fungi often form symbiotic relationships with various plant families, including Dipterocarpaceae, Fabaceae, Myrtaceae, Fagaceae, and Pinaceae, underscoring their ecological significance (Han *et al*. 2020). There are approximately 40 taxa within the genus *Strobilomyces*, and most species appear to be geographically restricted, exhibiting a high level of endemism (Han *et al*. 2018). One species, *Strobilomyces alpinus* Zang 1985 stands out as a potentially valuable edible fungus (Zang 1985). It is native to subalpine regions of southwestern and central China, and is often found growing solitarily or scattered on the soil or trunks of trees in forests dominated by *Abies* species. Its endemicity and distinct ecological niche, particularly its preference for *Abies* spp. as hosts, along with the largest basidiospores (11.5–14 × 9.5–11 μm) among all *Strobilomyces* species, indicate that *S. alpinus* represents a unique species within the genus (Han *et al*. 2020). Consequently, it has been included in China's red list of macrofungal biodiversity (https://www.mee.gov.cn/xxgk2018/xxgk/xxGk01/201805/W020180926382630924936.pdf), highlighting its uncertain population distribution and risk of extinction.

The advent of high-throughput sequencing technology has significantly advanced the field of mycology, enabling the sequencing of an increasing number of fungal genomes (Priest *et al*. 2020; Zhang *et al*. 2022; Xu *et al*. 2023). The genomes of macrofungal species exhibit remarkable variability in size, ranging from 27.4 to 202.2 Mb, and encode between 9,511 and 52,289 protein-coding genes (Li *et al*. 2018). These advancements have been particularly impactful in the study of macrofungal genomes, which often exceed 100 Mb in size and display considerable diversity in complexity. Comparative genomics has further facilitated the identification of conserved and unique genetic features across diverse fungal species, illuminating aspects of fungal phylogeny, classification, and the evolution of virulence and pathogenicity (Xie *et al*. 2024). The genomes of macrofungi are of paramount importance in both agriculture and medicine (Yu *et al*. 2022), offering a wide range of benefits to human health (Shao *et al*. 2020; Bhambri *et al*. 2022; Gebreyohannes *et al*. 2023). Some species within the Boletaceae have been sequenced and analyzed. Miyauchi *et al*. (2020) conducted a combined analysis of 135 mycorrhizal fungal genomes, including species from the Boletaceae, revealing the genomic changes associated with the transition from saprotrophy to symbiosis. Utilizing a dataset of 1,764 single-copy genes, Tremble *et al*. (2024) conducted a comprehensive and data-rich molecular phylogenetic analysis of the Boletaceae. They compared the genomes of 21 symbiotrophic species with their saprotrophic brown-rot relatives, identifying genomic traits that facilitate the transition to ectomycorrhizal ecology within the Boletales (Wu *et al*. 2021).

The genus *Strobilomyces* is distinguished by its rich species diversity, variable morphological characteristics, and significant ecological importance (Han *et al*. 2019; Deng *et al*. 2023). To date, notable research achievements have been made in the areas of systematics, classification, origin, and evolution, providing a crucial scientific foundation for a deeper understanding of the genus *Strobilomyces* (Han *et al*. 2018, 2020; Liu *et al*. 2025). To elucidate the genetic and physiological underpinnings of *S. alpinus*, whole-genome sequencing was performed using PacBio SMRT sequencing technology. Concurrently, a comparative analysis was conducted with the genomes of other closely related fungi, aiming to furnish genomic data for further research on the evolutionary aspects and biological functions of *S. alpinus.*

## Materials and methods

### Fungal strains and nucleic acid extraction

**Fig. 1.**
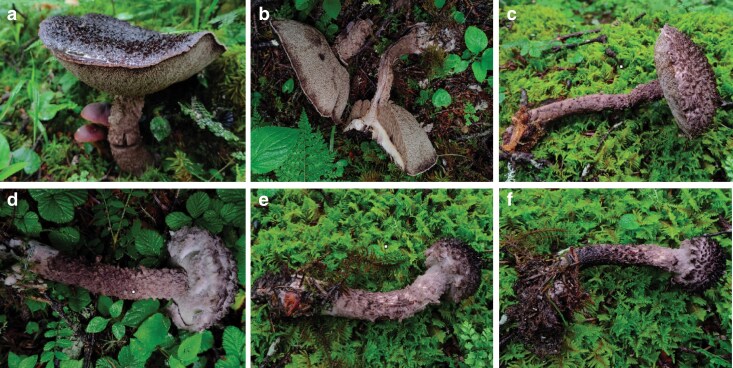
Fruiting bodies of *S. alpinus*. (a–b) Mature fruiting bodies. (c–f) Immature fruiting bodies.

The fruiting bodies of wild *S. alpinus* were collected from the White Horse Snow Mountain in Deqin County, Yunnan Province, China. One specimen, identified through morphological and molecular analyses conducted by L.-H.H., has been archived in the herbarium of Biological Resources and Food Engineering at Qujing Normal University, with the accession number 2022Sgenome1 (Fig. 1).

The genomic DNA of *S. alpinus* was extracted from freeze-preserved tissue using a modified CTAB method (Doyle and Dickson 1987). The integrity of the DNA was evaluated with a NanoDrop (Thermo Fisher Scientific, USA), yielding OD260/280 ratios between 1.8 and 2.0. Subsequently, a Qubit 3.0 Fluorometer (Invitrogen, USA) was employed for precise quantification of the DNA. Total RNA was extracted from the fruiting body tissue using a Plant RNA Purification Reagent. The concentration and purity of the RNA were assessed with a NanoDrop 2000, while the RNA Integrity Number was determined using an Agilent 2100 Bioanalyzer.

### Genome sequencing and assembly

Sequencing libraries were constructed following the manufacturer's instructions for Pacific Biosciences Sequel II HiFi sequencing, using the P6 polymerase/C4 chemistry (Pacific Biosciences, USA). A total of 1.5 µg of genomic DNA (gDNA) was fragmented into 10-kb segments using Covaris g-Tubes. To assess the quality of the libraries, we employed an Agilent 2,100 Bioanalyzer (Agilent, Santa Clara, CA, USA) to analyze the size distribution. After completing the library construction, the DNA library, Sequel Sequencing Primer v2, and polymerase were mixed in a specific ratio using the Sequel II Binding Kit 2.0 on-machine kit. The library was then transferred into the nanopores of the Sequel II series sequencer to initiate real-time single-molecule sequencing. Subsequently, the raw data obtained from sequencing was filtered to yield high-quality DNA sequence data. For the Sequel off-machine data, we utilized the built-in high-quality region finder (HQRF) in SMRTLink software, operating with default parameters, to identify the longest region of singly loaded enzyme activity. The software's default filtering, based on Signal Noise Ratio, automatically designates reads that meet its internal quality criteria as high-quality. After filtering, all resulting data have a read quality value of 0.8 or higher. Approximately 8.5 Gb of sequencing data were produced, comprising 472,906 high-quality HiFi reads, with an average read length of 18,129 bp (Table 1). For genome assembly, we employed Hifiasm software (v0.15.4) (Cheng *et al*. 2021) with its default settings, utilizing the high-fidelity (HiFi) reads. Additionally, we performed RNA sequencing using the DNBSEQ-T7 sequencer, with a read length of 2 × 150 bp. Total RNA was extracted from freeze-preserved tissue following the manufacturer's protocol. From the total RNA, poly-A RNAs were enriched, fragmented, and cDNA was synthesized. After end-repair and other necessary steps, libraries were PCR-amplified, purified, analyzed, circularized, and sequenced.

**Table 1.** Summary of the *S. alpinus* genome assembly and annotation features.

Type	Value
Genome size (Mb)	58.75
Pair-end libraries	PacBio Sequel II
Total reads (#)	472,906
Total base (Gb)	8.57
Average coverage depth (X)	111.6
Average length of reads (bp)	18,129
Genome N50 (Mb)	4.03
Contig number	68
Contig reads GC (%)	54.87
Shortest contig (bp)	25,546
Longest contig (bp)	5,320,747
Complete Buscos (%)	96.06
Gene numbers	11,761
Average gene length (bp)	2480.88
Average CDS length (bp)	1301.2
Average number of exons per gene	6.2
Average exon length (bp)	209.94
Average intron length (bp)	226.95

### Genome quality assessment

To evaluate the functional completeness of our genome assembly, we employed Benchmarking Universal Single-Copy Orthologs (BUSCO) v5.2.2 (Manni *et al*. 2021). We utilized Merqury v20200318 (Rhie *et al*. 2020) to estimate the k-mer completeness of the raw HiFi reads and to assess the base error rates of the assembly.

### Genomic component analysis

#### Repeat sequence prediction

In this study, we initially annotated tandem repeats using the software GMATA (Wang and Wang 2016) and Tandem Repeats Finder (TRF) (Benson 1999). Specifically, GMATA was employed to identify simple sequence repeats (SSRs), while TRF detected all tandem repeat elements across the entire genome. Subsequently, we integrated both ab initio and homology-based approaches to uncover transposable elements (TEs) in the *S. alpinus* genome. To begin, we predicted an ab initio repeat library specific to *S. alpinus* using MITE-hunter (Han and Wessler 2010) and RepeatModeler with default parameters. This library generation process also incorporated tools such as LTR_FINDER, LTR_harvest, and LTR_retriever, which are specifically designed for plant genomes. The resulting library was then aligned with the TEclass Repbase (http://www.compgen.uni-muenster.de/teclass/) for the classification of each repeat family. We utilized RepeatMasker (Bedell *et al*. 2000) to further identify TEs throughout the genome. Finally, overlapping TEs belonging to the same repeat class were compiled and consolidated.

#### Protein-coding gene prediction

Three independent approaches were employed for gene prediction: ab initio prediction, homology search, and reference-guided transcriptome assembly. GeMoMa (Keilwagen *et al*. 2019) was utilized to align homologous peptides from related species to the *S. alpinus* genome, thereby obtaining gene structure information through homologous prediction. For RNA-seq-based gene prediction, filtered mRNA-seq reads were aligned to the reference genome using STAR. The transcripts were subsequently assembled using StringTie (Kovaka *et al*. 2019), and open reading frames (ORFs) were predicted with PASA (Haas *et al*. 2008). In the de novo prediction approach, RNA-seq reads were assembled de novo using StringTie and analyzed with PASA to create a training set. Augustus (Stanke *et al*. 2008) was then employed with default parameters for ab initio gene prediction. Finally, EVidenceModeler (EVM) (Haas *et al*. 2008) was utilized to generate an integrated gene set, from which genes associated with TEs were removed using the TransposonPSI package (http://transposonpsi.sourceforge.net/), and incorrectly annotated genes were further filtered out. Untranslated regions (UTRs) and alternative splicing regions were identified using PASA based on RNA-seq assemblies. We retained the longest transcripts for each locus, designating regions outside of the ORFs as UTRs.

#### Genome functional annotation

Gene function information, as well as the motifs and domains of their proteins, was assigned by comparing them with public databases, including SwissProt, Non-Redundant (NR), the Kyoto Encyclopedia of Genes and Genomes (KEGG), Eukaryotic Orthologous Groups of proteins (KOG), and Gene Ontology (GO). The putative domains and GO terms of the genes were identified using the InterProScan program with default parameters. For the other 4 databases, BLASTp was employed to compare the EVM-integrated protein sequences against these well-known public protein databases, using an E-value cutoff of 1e-05. The results with the lowest E-value were retained. The results from the 5 database searches were then concatenated.

#### Secretome prediction

Carbohydrate-active enzymes (CAZymes) in *S. alpinus* were annotated and classified using the dbCAN annotation program (https://bcb.unl.edu/dbCAN2/blast.php). Candidate pathogen-host interaction (PHI) genes within the genome of *S. alpinus* were identified by employing BLASTP to search against PHI-base v4.3. The analysis of cytochrome P450 (CYP) monooxygenases was conducted by querying a reference dataset with the BLASTP program. The prediction of biosynthetic gene clusters (BGCs) in *S. alpinus* was performed using the fungal version of antiSMASH (https://fungismash.secondarymetabolites.org), with the detection strictness set to relaxed and the extra features parameter enabled.

#### Annotation of non-coding RNAs

Two strategies were employed to obtain non-coding RNA (ncRNA): database searching and model prediction. Transfer RNAs (tRNAs) were predicted using tRNAscan-SE with eukaryotic parameters. MicroRNA (miRNA), ribosomal RNA (rRNA), small nuclear RNA (snRNA), and small nucleolar RNA (snoRNA) were identified using Infernal cmscan (Nawrocki and Eddy 2013) to search the Rfam database. The rRNAs and their subunits were predicted using RNAmmer (Lagesen *et al*. 2007).

### Gene expression analysis

The transcriptome data was filtered using fastp (Chen *et al*. 2018) with default parameters to remove low-quality reads and adapter sequences. The filtered transcriptome data was then aligned to the genome using STAR (Dobin *et al*. 2013), also with default parameters, to generate an alignment file. The resulting SAM file was subsequently converted to BAM format using samtools (Li *et al*. 2009). Based on the sorted BAM file, StringTie (Kovaka *et al*. 2019) was employed to calculate Fragments per Kilobase Million (FPKM) values. Finally, the FPKM values of the annotated genes were summarized. A gene was considered to have transcript support if its FPKM value was greater than zero.

### Comparative genomic analysis

Interspecific and intraspecific genomic collinearity analyses were conducted on the protein sequences of *Boletus edulis* and *Boletus reticuloceps* in relation to *S. alpinus*. Genome data for the former 2 species were obtained from the National Center for Biotechnology Information (https://www.ncbi.nlm.nih.gov/). MCScanX (Wang *et al*. 2012) was employed to identify the intragenome collinear blocks of the 3 species. Additionally, the predictive BGCs of the intraspecific genomic collinearity analysis were visualized using Circos (Krzywinski *et al*. 2009). The genomic collinearity relationships among the 3 species were illustrated using TBtools software (Chen *et al*. 2020).

## Results and discussion

### Genome assembly and evaluation

By integrating the sequences of ITS, *ef1-α*, *rpb1*, and *rpb2* with morphological analysis (Fig. 1), the *Strobilomyces* strain 2022Sgenome1 was identified as *S. alpinus*. This species displayed a black-brown to black-purple pileus, with rusty red discoloration of the context upon exposure, and demonstrated a strong preference for associating with *Abies* spp.

A total of 8.57 Gb of PacBio HiFi data were obtained, comprising 472,906 total reads, using the Pacific Biosciences Sequel platform. The average read length was 18.13 kb, with an N50 length of 4.03 Mb. After quality control, the assembled genome of *S. alpinus* measured 58.75 Mb, with a GC content of 54.87% (Table 1). The average sequencing depth was 111.6, consisting of 68 contigs, with the longest sequence measuring 5,320,747 bp and the shortest sequence measuring 25,546 bp. Clean data from the DNBSEQ-T7 platform were aligned to the assembled genome to assess the quality of the genome assembly. The mapping rate of the transcriptome data was 93.15%. The assessment of genome integrity using BUSCO analyses yielded a score of 93.17% (complete and single-copy BUSCOs: 91.21%, complete and duplicated BUSCOs: 1.97%, fragmented BUSCOs: 0.14%, and missing BUSCOs: 6.68%) (Supplementary Table 1). This indicates that the vast majority of the conserved core genes of fungi were predicted, suggesting that the assembly of the *S. alpinus* genome is of high quality. This high-quality assembly provides a solid foundation for subsequent comparative genomic analyses, allowing us to explore the evolutionary relationships, shared and unique genetic features, and potential adaptive mechanisms of *S. alpinus* in comparison to its close relatives.

### Genomic collinearity analysis

**Fig. 2.**
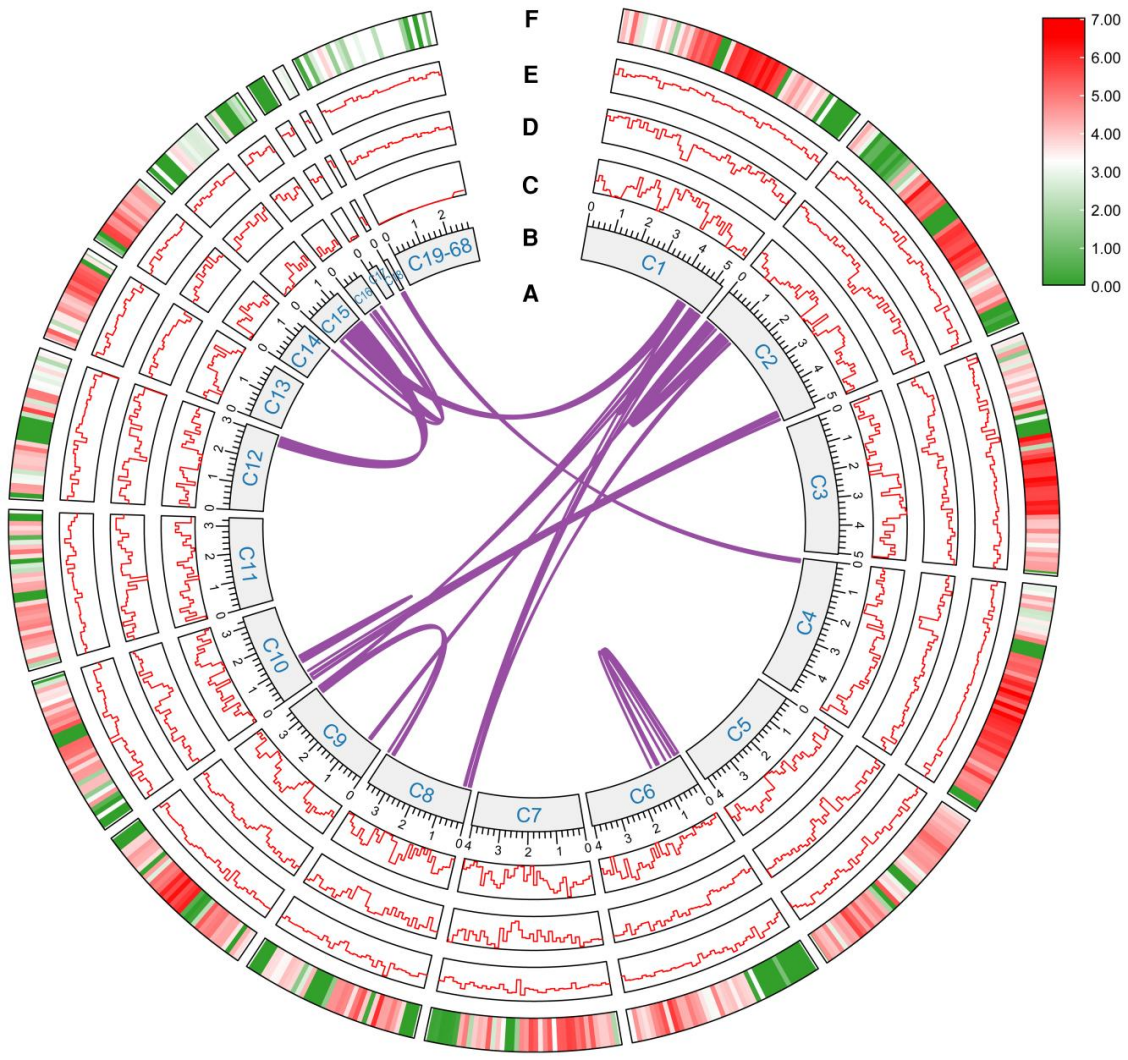
Genomic features of *S. alpinus*. The figure presents several genomic analyses arranged from the innermost to the outermost circle: genomic collinearity analyses (a), a contig composition map (b), a line chart depicting gene density (c), a line chart illustrating GC content (d), a line chart showing GC skew (e), and a heatmap of gene expression (f). The gene expression values were normalized and log2-transformed from FPKM.

The genomic collinearity analyses were conducted on 68 contigs of *S. alpinus*, and the essential genomic features were incorporated into the circular genome map (Fig. 2). The collinearity graph reveals no evidence of whole-genome or segmental duplications in *S. alpinus*.

The genome size of mycorrhizal fungi is larger than that of saprotrophic species (Miyauchi *et al*. 2020). According to publicly available information, the genome size of fungi in the Boletaceae family ranges from 33.98 to 69.19 Mb (Wu *et al*. 2021). When compared with similar species of *Boletus*, the genome size of *S. alpinus* is larger than that of *B. coccyginus* (54.9 Mb) and *B. reticuloceps* (55.8 Mb), but smaller than that of *B. edulis* (66.5 Mb) (Table 2). The number of contigs and the contig N50 indicate that we achieved a superior genome assembly of *S. alpinus* in this study. The GC content in the *S. alpinus* genome (54.87%) is higher than that in the *B. edulis* genome (50.5%) and the *B. reticuloceps* genome (50%). Additionally, the average transcript length, average coding sequence (CDS) length, and average number of exons per gene in *S. alpinus* are greater than those in the *B. edulis* and *B. reticuloceps* genomes (Table 2).

**Table 2.** Comparison of the *S. alpinus* genome with the gene sets of closely related species.

Type	*S. alpinus*	*B. edulis*	*B. reticuloceps*
Assembly accession	2022Sgenome1	GCA_015179015.1	GCA_018397855.1
Genome size (Mb)	58.75	66.5	55.8
Number of scaffolds	68	593	126
Contig N50	4.03 Mb	420.5 kb	1.1 Mb
GC percent (%)	54.87	50.5	50
Total number of genes	11,761	18,355	13,195
Average transcript length (bp)	2,480.88	1,350.34	1,479.34
Average CDS length (bp)	1,301.20	1,083.38	1,226.76
Average exons number per gene	6.2	5.07	5.07
Average exon length (bp)	209.94	213.53	241.93
Average intron length (bp)	226.95	65.54	62.05

**Fig. 3.**
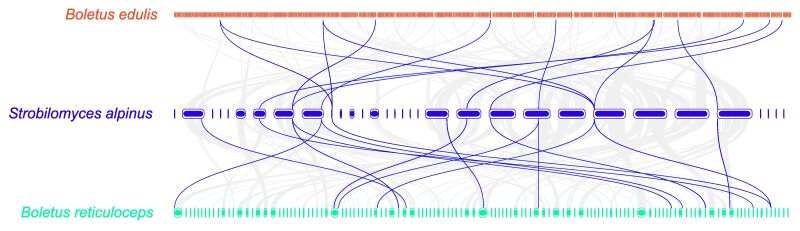
Genomic collinearity analysis among *S. alpinus*, *B. edulis*, and *B. reticuloceps*. The bold line indicates the gene clusters associated with secondary metabolites.

The genomic collinearity analyses among *S. alpinus*, *B. edulis,* and *B. reticuloceps* were conducted separately, revealing a high degree of consistency among all 3 species (Fig. 3). This consistency can be attributed to their common ancestry within the Boletaceae. Furthermore, the observed dispersion of genes in the 2 species of the genus *Boletus* may result from challenges encountered during genome assembly.

### Evaluation of genome annotation

The predicted gene set was evaluated using BUSCO, which assesses the assembly integrity of the existing genome sequence by utilizing conserved single-copy homologous genes from the boletales_odb10 library. Approximately 93.17% of the complete gene elements are present in the annotated gene set, indicating that the majority of conserved gene predictions are relatively complete. This finding indirectly reflects the high reliability of the prediction results (Supplementary Table 1). A total of 11,761 genes were predicted in the *S. alpinus* genome (Table 1), with an average gene length of 2,480.88 bp and an average CDS length of 1,301.2 bp. Each gene contains an average of 6.2 exons, with an average exon length of 209.94 bp and an average intron length of 226.95 bp (Table 1, Supplementary Fig. 1). Additionally, the non-coding RNAs (ncRNAs) in the *S. alpinus* genome include 532 rRNA, 62 small RNAs, 98 tRNAs, and 1 regulatory RNA (Supplementary Table 2).

### Repeat sequence annotation

Transposable element proliferation has significantly contributed to the increased evolutionary rate of genes encoding effector-like small secreted proteins, proteases, and lipases (Wu *et al*. 2021). A total of 50,700 repetitive sequences were identified, encompassing 44.28% of the entire genome, with a cumulative length of 26,014,302 bp in the *S. alpinus* genome (Supplementary Table 3). Specifically, TEs account for 37.14% of the genome, with long terminal repeats (LTRs), DNA transposons, long interspersed nuclear elements (LINEs), and short interspersed nuclear elements (SINEs) representing approximately 27.15, 5.51, 3.78, and 0.13%, respectively. The proportion of tandem repeats is 1.02%, with tandem repeats and SSRs comprising 0.99 and 0.03%, respectively. The proportions of simple repeats and unknown repeat sequences are 0.46 and 5.52%, respectively. Additionally, *S. alpinus* contains 15.95 megabase pairs of LTRs, including Gypsy, Copia, and unclassified LTR elements, which are the dominant TEs and occupy 25.54% of the genome. The distribution of TE categories varies among ectomycorrhizal fungal taxa, with Gypsy and Copia LTRs being particularly abundant in Basidiomycota (Miyauchi *et al*. 2020).

### Functional annotation of the genome

Annotation was conducted using the NCBI NR, KEGG, GO, KOG, and SwissProt databases (Table 3). A total of 10,808 genes were annotated from these public databases, representing 91.90% of the overall gene count. Among the annotated genes, 10,805 were identified in the NR database, followed by 6,157 in SwissProt, 5,259 in GO, 4,064 in KEGG, and 3,098 in KOG (Table 3). Among the 9 genome sequences of Boletaceae fungi that are both publicly available and fully annotated, the number of genes ranges from 12,051 to 18,123 (Miyauchi *et al*. 2020; Wu *et al*. 2021). In comparison, *S. alpinus* contains 11,761 genes, which is slightly fewer than those found in other species of the Boletaceae.

**Table 3.** Summary of gene annotations for *S. alpinus* from public databases.

Type		Number	Percent (%)
Annotation	SwissProt	6,157	52.35
	KEGG	4,064	34.55
	KOG	3,098	26.34
	GO	5,259	44.72
	NR	10,805	91.87
Secreted protein	protein with signal peptide	551	4.68
	protein without transmembrane domain	9,873	83.95
	secreted protein	382	3.25
Functional proteins	CAZy	813	6.91
	PHI	1,821	15.48
	P450	1,938	16.48
Total	Annotation	10,808	91.9
	Gene	11,761	–

KEGG, Kyoto Encyclopedia of Genes and Genomes; KOG, Eukaryotic Orthologous Groups; GO, Gene Ontology; NR, National Center for Biotechnology Information Non-Redundant Protein Database; PHI, Pathogen–Host Interactions Database; CAZy, Carbohydrate-Active Enzymes Database; P450, Cytochrome P450 Monooxygenase Database.

As secreted proteins play a crucial role in the decomposition of soil organic matter and the development of symbiosis, we predicted the secretomes within the *S. alpinus* genome. A total of 382 coding genes for secreted proteins were identified, representing 3.25% of the overall protein repertoire. The proportion of secreted proteins is 4.6–5.7% of the total protein repertoire for Basidiomycota and Ascomycota, respectively (Miyauchi *et al*. 2020). No expansion of gene families coding for secreted proteases, phosphatases, or phytases was observed in ectomycorrhizal fungi (Miyauchi *et al*. 2020).

The non-redundant proteins annotated in *S. alpinus* exhibited the closest matches with *Imleria badia* (3,704 proteins, 34.28%), *B. edulis* (2,336 proteins, 21.62%), *B. coccyginus* (1,443 proteins, 13.35%), *B. reticuloceps* (1,109 proteins, 10.26%), *Lanmaoa asiatica* (696 proteins, 6.44%), *Butyriboletus roseoflavus* (468 proteins, 4.33%), and *Chiua virens* (267 proteins, 2.47%). Collectively, these accounted for 92.76% of the total non-redundant predicted genes (Supplementary Fig. 2).

**Fig. 4.**
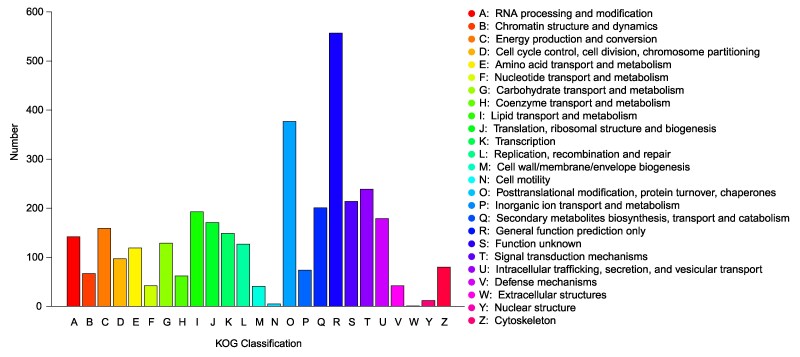
Classification chart of annotated results from the KOG database.

KOG is a gene orthology database for eukaryotes. In this study, 3,479 genes were assigned to the KOG categories (Fig. 4). The majority of the genes were classified into the “General function prediction only” category (557), followed by “Posttranslational modification, protein turnover, chaperones” (377), “Signal transduction mechanisms” (239), and “Secondary metabolites biosynthesis, transport, and catabolism” (201). Fewer genes were categorized under “Extracellular structures” (1), “Cell motility” (5), and “Nuclear structure” (12). The representation of genes related to posttranscriptional regulation and signal transduction may reflect the capacity of *S. alpinus* to adapt to its environment. The quantity and distribution of KOG categories are very similar to those of *Ganoderma leucocontextum* (Liu *et al*. 2021) and *Haromycopsis phalluae* (Yuan *et al*. 2021). In contrast, *Naematelia aurantialba* (Sun *et al*. 2021) and *Sanghuang* (Shao *et al*. 2020) exhibit a higher proportion of genes in the categories of “Translation, ribosomal structure, and biogenesis” and “Energy production and conversion.”

**Fig. 5.**
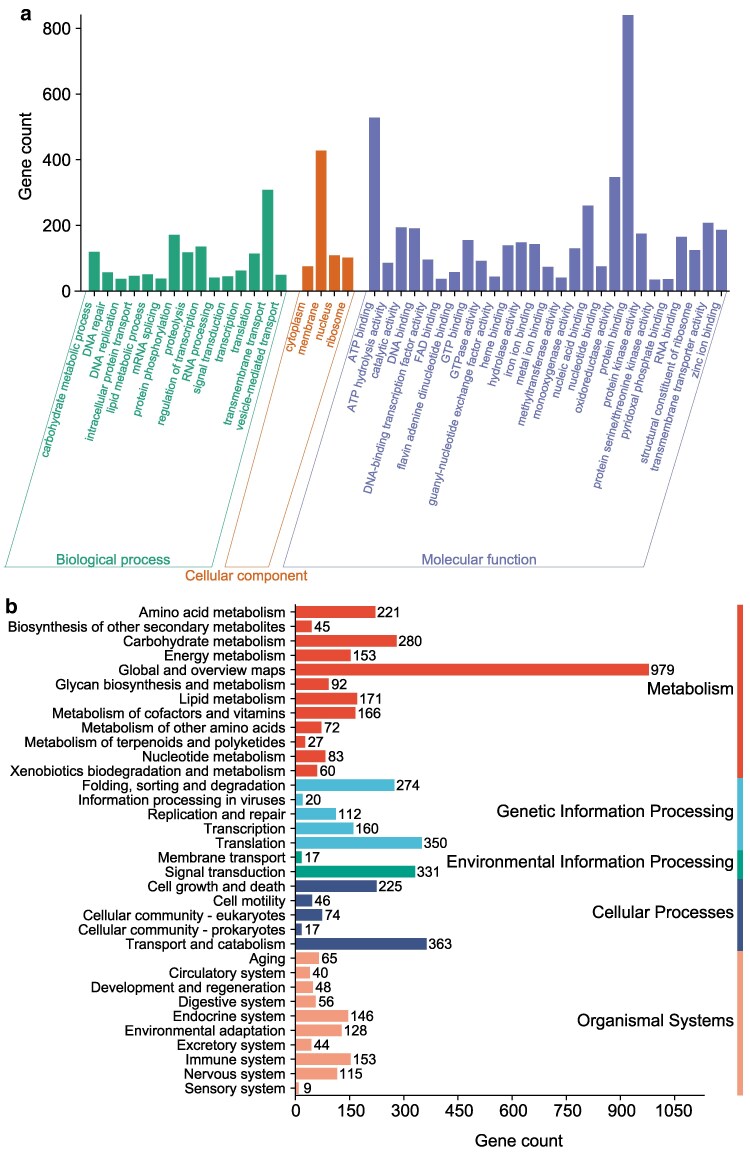
Classification statistics for GO (a) and KEGG (b) annotations of the *S. alpinus* genome.

In terms of GO functional classes, a total of 1,913 GO annotations were identified, corresponding to 5,259 proteins, which represent 44.72% of the total predicted proteins. These proteins were categorized into 3 functional categories. The molecular function category was predominantly represented by “protein binding” (840), “ATP binding” (528), and “oxidoreductase activity” (347). The cellular components primarily included “membrane” (428). Among the biological processes, “transmembrane transport” (308) contained the highest number of proteins (Fig. 5a).

KEGG is a comprehensive database that compiles information on the genomes, pathways, and compounds of various organisms, enhancing our understanding of gene functions in *S. alpinus*. According to the results of KEGG functional annotation, 5,142 genes were categorized into 5 physiological processes: “Organismal Systems” (804), “Metabolism” (2,349), “Cellular Processes” (725), “Genetic Information Processing” (916), and “Environmental Information Processing” (348) (Fig. 5b). In the second layer of KEGG pathway terms, the metabolism pathway associated with “Global and Overview Maps” (979) contained the highest number of genes. For cellular processes, “Transport and Catabolism” (363) had the most genes involved, while for genetic information processes, “Translation” (350) was the most prominent. In terms of environmental information processes, “Signal Transduction” (331) had the highest involvement (Fig. 5b). These findings may elucidate the genetic basis for the richness of signal response and metabolic processes in *S. alpinus*.

### Annotation of carbohydrate-active enzymes

**Fig. 6.**
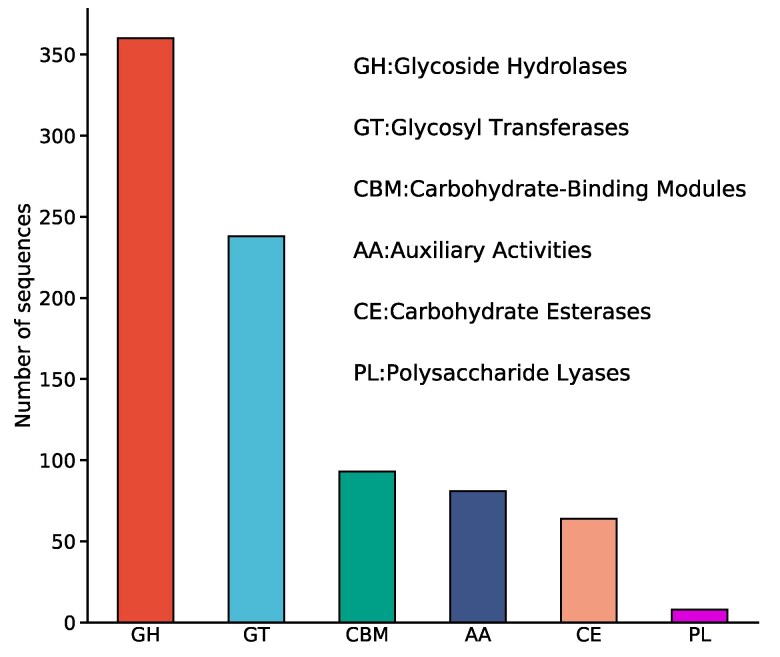
CAZy functional annotation of *S. alpinus* genes.

The CAZymes play crucial roles in the degradation of lignocellulose, providing carbohydrates essential for fungal growth, development, and stress responses (Xie *et al*. 2024). The CAZy database was utilized to map the genome of *S. alpinus* to investigate the distribution of CAZymes. A total of 813 CAZyme-coding genes were annotated (Table 3), which included 348 glycoside hydrolases (GHs), 231 glycosyltransferases (GTs), 82 carbohydrate-binding modules (CBMs), 80 auxiliary activities (AAs), 64 carbohydrate esterases (CEs), and 8 polysaccharide lyases (PLs) (Fig. 6). In the second layer of category terms, AA1 (33) and AA3 (14) were the most prevalent categories, involved in lignin and cellulose degradation, respectively. The families CBM50 (18) and CBM13 (15) were also abundant. GHs accounted for 42.80% of the total identified CAZymes in *S. alpinus*, with GH18 (32) being the most represented category, which hydrolyzes the β-1,4-linkages in chitin. Proteins in the CE category were primarily distributed among the CE3 and CE4 families, which remove acetyl groups in xylan and enhance its accessibility to xylanase. GT4 (35) and GT2 (33) were the 2 most abundant glycosyltransferase categories.

The polyphyletic evolution of the ectomycorrhizal lifestyle is characterized by the convergent and nearly complete loss of core cellulose- and lignin-degrading CAZymes. However, many ectomycorrhizal symbionts studied have retained a unique array of plant cell-wall-degrading enzymes (PCWDEs), including endoglucanases (GH5), pectinases (GH28), and oxidoreductases/laccases (AA1, AA9). This retention suggests that these organisms possess diverse capabilities to scavenge plant and microbial detritus from soil and litter (Wu *et al*. 2021). The loss of cellobiohydrolase GH6, cellobiohydrolase GH7, and endoglucanases (CBM1-GH5_5) coding genes in ectomycorrhizal Boletales is associated with the accumulation of discrete nucleotide mutations, a phenomenon known as the DNA decay process (Wu *et al*. 2021).

Ectomycorrhizal Boletales contain significantly fewer secreted PCWDEs compared with their brown-rot relatives (Wu *et al*. 2021). Notably, their genome lacks the invertase GH32 gene, suggesting that ectomycorrhizal basidiomycetes are unable to directly utilize sucrose from plants (Miyauchi *et al*. 2020). Ectomycorrhizal fungi have generally retained genes for microbial cell wall-degrading enzymes, such as chitinases and β-1,3-glucanases, although they have lost most plant cell wall-degrading enzymes, including endo- and exocellulases. Compared with other Boletaceae fungi, *S. alpinus* possesses the highest number of genes in each category of CAZymes. GHs, GTs, and AAs—which catalyze the modification, biosynthesis, or degradation of glycoconjugates and carbohydrates—are the predominant CAZymes in the *S. alpinus* genome. CMBs are non-catalytic modules that are appended to these enzymes. *S. alpinus* has a significantly higher number of candidate CAZymes than other edible fungi (Kiyama *et al*. 2018), indicating its potential for more efficient utilization of hardwood. Therefore, *S. alpinus* may be classified as a fungus with exceptionally well-developed carbohydrate utilization capabilities.

CYP is a superfamily of hemoproteins that utilize heme as a cofactor. These enzymes exhibit a diverse array of substrates across various enzymatic reactions and are present in all biological kingdoms. Through a BLAST search with an *E*-value of less than 1e^−5^, we identified 1,938 putative CYP superfamily genes in *S. alpinus* (Supplementary Table 4).

**Fig. 7.**
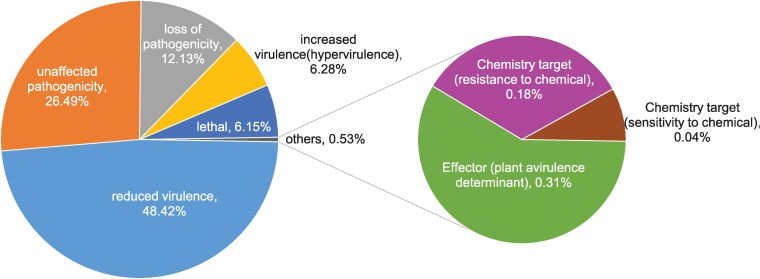
Functional annotation of *S. alpinus* genes using PHI.

Amino acid sequences were analyzed using PHI-base, leading to the identification of 1,821 candidate pathogenicity-related proteins (Fig. 7). The “Reduced Virulence” category contained the highest number of enriched proteins (1,102), followed by “Unaffected Pathogenicity” (603), and “Loss of Pathogenicity” (276), these categories accounted for 87.04% of all proteins predicted by PHI-base.

### Genomic analyses of antiSMASH annotations

Considering the potential application value of its antimicrobial activities, we further investigated the BGCs of secondary metabolites in *S. alpinus* building on those identified in previous studies. Based on antiSMASH predictions, we identified 16 secondary metabolite gene clusters in the *S. alpinus* genome, which include 7 NRPS-like clusters, 5 terpene clusters, 1 fungal RiPP-like cluster, 1 RiPP-like cluster, 1 T1PKS cluster, and 1 T1PKS-NRPS linkage gene cluster (Supplementary Table 5). These genes are distributed across 12 contigs (Fig. 3). Unlike primary metabolism, secondary metabolism does not contribute to the growth and development of an organism; rather, it enhances survival in a given environment, for example, by strengthening the defense response to pathogens.

Terpene synthases are versatile catalysts found in all domains of life, facilitating the formation of a diverse array of terpenoid secondary metabolites. The sesquiterpene (+)-δ-cadinol has previously demonstrated cytotoxic activity, indicating its potential as a new, sustainably sourced anti-tumor agent (Ringel *et al*. 2022). The therapeutic efficacy of terpenes derived from mushrooms has been well established (Duru and Çayan 2015). Five candidate gene clusters were identified in contigs 6, 9, 11, and 13, exhibiting at least 85% identity at the amino acid sequence level. Given the conserved structure of armillyl orsellinate, we identified a candidate gene cluster located in contig 11 (positions 2,981,268–3,025,298), which catalyzes the synthesis of armillyl orsellinate from 8α-hydroxy-6-protoilludene (Engels *et al*. 2020).

### Biosynthesis of bioactive compounds

#### Terpenoid backbone biosynthesis

**Fig. 8.**
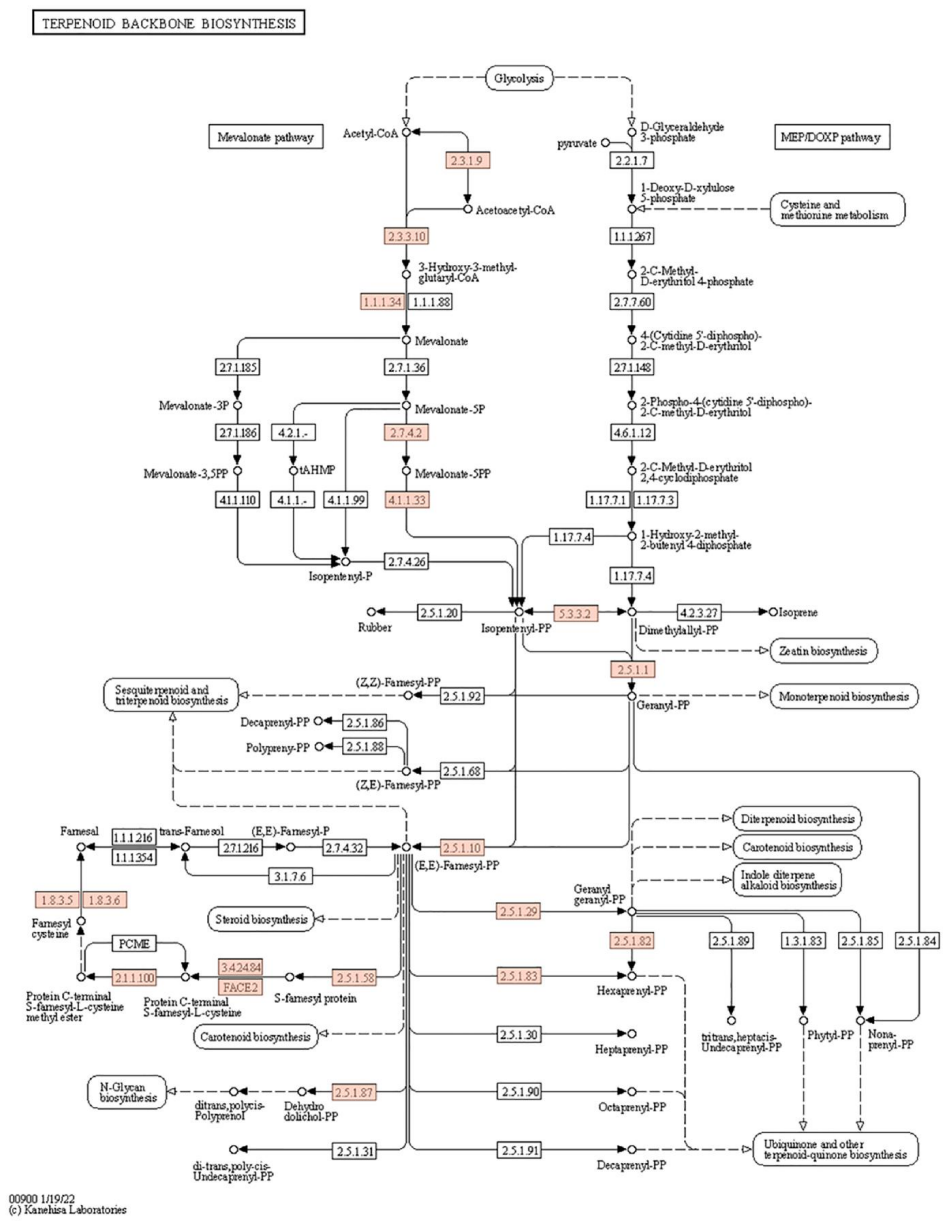
KEGG mapping of the terpenoid backbone biosynthesis pathway in *S. alpinus.*

Terpenoids, the largest family of natural products, are essential components of human medicine (Luo *et al*. 2024). A total of 15 key enzymes are involved in the mevalonate pathway (Fig. 8). The proteins farnesyltransferase subunit beta, ditrans-polycis-polyprenyl diphosphate synthase, geranylgeranyl diphosphate synthase, farnesyl diphosphate synthase, and geranyl diphosphate synthase are each encoded by 2 or 3 copies of their respective genes (Table 4). Gene *Contig9g00780* encodes farnesyl diphosphate farnesyltransferase, while another gene, designated *mRNA3556151*, encodes squalene monooxygenase, which catalyzes the biosynthesis of sesquiterpenoids and triterpenoids (Supplementary Fig. 3). Fungi, such as *Strobilomyces*, have emerged as significant sources of diverse hybrid terpenoid natural products, and elucidating their biosynthetic pathways will facilitate the development of potential drug candidates in the future (Yuan *et al*. 2024).

**Table 4.** Enzymes involved in terpenoid backbone biosynthesis in *S. alpinus*.

Entry	Enzyme	Enzyme ID	ID
K00626	Acetyl-CoA C-acetyltransferase	EC:2.3.1.9	*Contig3g00815*
K01641	Hydroxymethylglutaryl-CoA synthase	EC:2.3.3.10	*Contig9g00566*
K00021	Hydroxymethylglutaryl-CoA reductase	EC:1.1.1.34	*Contig2g00971*
K00938/K13273	Phosphomevalonate kinase	EC:2.7.4.2	*Contig2g00333*
K01597	Diphosphomevalonate decarboxylase	EC:4.1.1.33	*Contig2g00971*
K01823	Isopentenyl-diphosphate Delta-isomerase	EC:5.3.3.2	*mRNA2943859*
K00787/K00795/ K00804/K13787/ K13789/K14066	Farnesyl diphosphate synthase Geranyl diphosphate synthase	EC:2.5.1.1 EC:2.5.1.10	*Contig11g00458* *Contig5g00140* *Contig5g00163*
K05906	Prenylcysteine oxidase Farnesylcysteine lyase	EC:1.8.3.5 EC:1.8.3.6	*Contig4g00398*
K00587	Protein-*S*-isoprenylcysteine *O*-methyltransferase	EC:2.1.1.100	*Contig3g00695*
K06013	STE24 endopeptidase	EC:3.4.24.84	*Contig11g00141*
K08658	Intramembrane prenyl-peptidase	EC:3.4.26.1	*Contig7g00639*
K05954/K05955	Protein farnesyltransferase subunit beta	EC:2.5.1.58	*Contig11g00118* *Contig6g00435*
K11778/K19177	Ditrans, polycis-polyprenyl diphosphate synthase	EC:2.5.1.87	*mRNA3477211* *Contig4g00766*
K00804/K13787/ K13789	Geranylgeranyl diphosphate synthase, type III	EC:2.5.1.1 EC:2.5.1.10 EC:2.5.1.29	*Contig5g00140* *Contig5g00163*
K05355/K21268/ K21274/K21275	Hexaprenyl diphosphate synthase	EC:2.5.1.82 EC:2.5.1.83	*mRNA2707596*

#### Porphyrin metabolism

Porphyrins are a class of macrocyclic compounds that play a crucial role in cancer diagnosis and treatment (Nishida *et al*. 2021). Their involvement in the generation, storage, and utilization of oxygen is essential for sustaining life. A total of 19 key enzymes participate in the biosynthesis of heme A and siroheme (Supplementary Fig. 4). Porphyrin compounds, which function as efficient cellular factories through enzymatic catalysis, have been produced using *Rhodobacter sphaeroides* (Senge *et al*. 2021). Several key enzyme genes have been identified in *S. alpinus*, which can be harnessed for porphyrin biosynthesis, offering significant industrial and medical applications.

#### Folate biosynthesis

Folates are essential for all living organisms. They are synthesized de novo in plants, bacteria, yeast, and fungi, serving as a crucial dietary source for animals (Gorelova *et al*. 2019). An analysis of 10 types of mushrooms indicates that they are a good source of folate (Phillips *et al*. 2011). However, the folate synthesis pathway in mushrooms is rarely explored. In this study, we present 22 genes related to folate synthesis in *S. alpinus* (Supplementary Fig. 5). The novel compositions of mushroom folate metabolism may pave the way for new directions in folate research.

## Conclusions

Here, we present the de novo assembled complete genome of *S. alpinus* for the first time. Integrity and genomic collinearity analyses revealed the high quality of the genome assembly and gene prediction within the Boletaceae. Based on the annotation of 11,761 protein-coding genes, *S. alpinus* demonstrated high annotation rates across the tested databases. This study indicates that *S. alpinus* has the potential to synthesize a variety of secondary metabolites, particularly genes involved in terpenoid biosynthesis, thereby providing important insights into the biological properties of this medicinal food fungus through whole-genome sequencing. Furthermore, the genome will facilitate research on the biosynthesis of medicinal compounds in *Strobilomyces* and offer valuable genetic resources for molecular breeding in edible *Strobilomyces* mushrooms. In conclusion, this study enriches the genetic database for the Boletaceae, enhances our understanding of its genomic structure and function, and lays a solid foundation for the future development and utilization of the genus *Strobilomyces*.

## Supplementary Material

jkaf080_Supplementary_Data

## Data Availability

Data for this study have been submitted to the National Center for Biotechnology Information (NCBI) under the BioProject number PRJNA1215829. The annotations of genome are available at Figshare (https://doi.org/10.6084/m9.figshare.28723994.v1). Concurrently, the same set of data has also been deposited to the China National Center for Bioinformation (https://ngdc.cncb.ac.cn) under the Bioproject number PRJCA032531. The online version contains Supplementary material available at G3 online. Supplementary Fig. 1: Characterization of distribution of CDS length (A), exon length (B), exon number (C), gene length (D), intron length (E), and intron number (F) in the genome of *S*. *alpinus* and 2 closely related species. Supplementary Fig. 2: The annotation classification of NR corresponding to *S. alpinus* to the other species; Supplementary Fig. 3: KEGG mapping of the sesquiterpenoid and triterpenoid biosynthesis pathways in *S. alpinus*; Supplementary Fig. 4: KEGG mapping of the porphyrin metabolism pathway in *S. alpinus*; Supplementary Fig. 5: KEGG mapping of the folate biosynthesis pathway in *S. alpinus*; Supplementary Table 1: The quality assessment for genome annotation of *S. alpinus*; Supplementary Table 2: Non-coding RNA annotation statistics for the genome of *S. alpinus*; Supplementary Table 3: Statistics of transposable element repeat sequences in *S. alpinus*; Supplementary Table 4: Putative CYP superfamily genes in *S. alpinus*; Supplementary Table 5: The putative genes involved in secondary metabolism of *S. alpinus*. Supplemental material available at G3 online.
