# Supplementary material for: Whole genome sequence of an edible mushroom Strobilomyces alpinus (Boletaceae)
Source: G3 (Bethesda). 2025 Apr 10;15(6):jkaf080. doi: 10.1093/g3journal/jkaf080 (PMC12135008; doi:10.1093/g3journal/jkaf080)
Supplement: jkaf080_Supplementary_Data [file jkaf080_supplementary_data.zip › Supplemental_Figures_G3-2025-405676.docx]

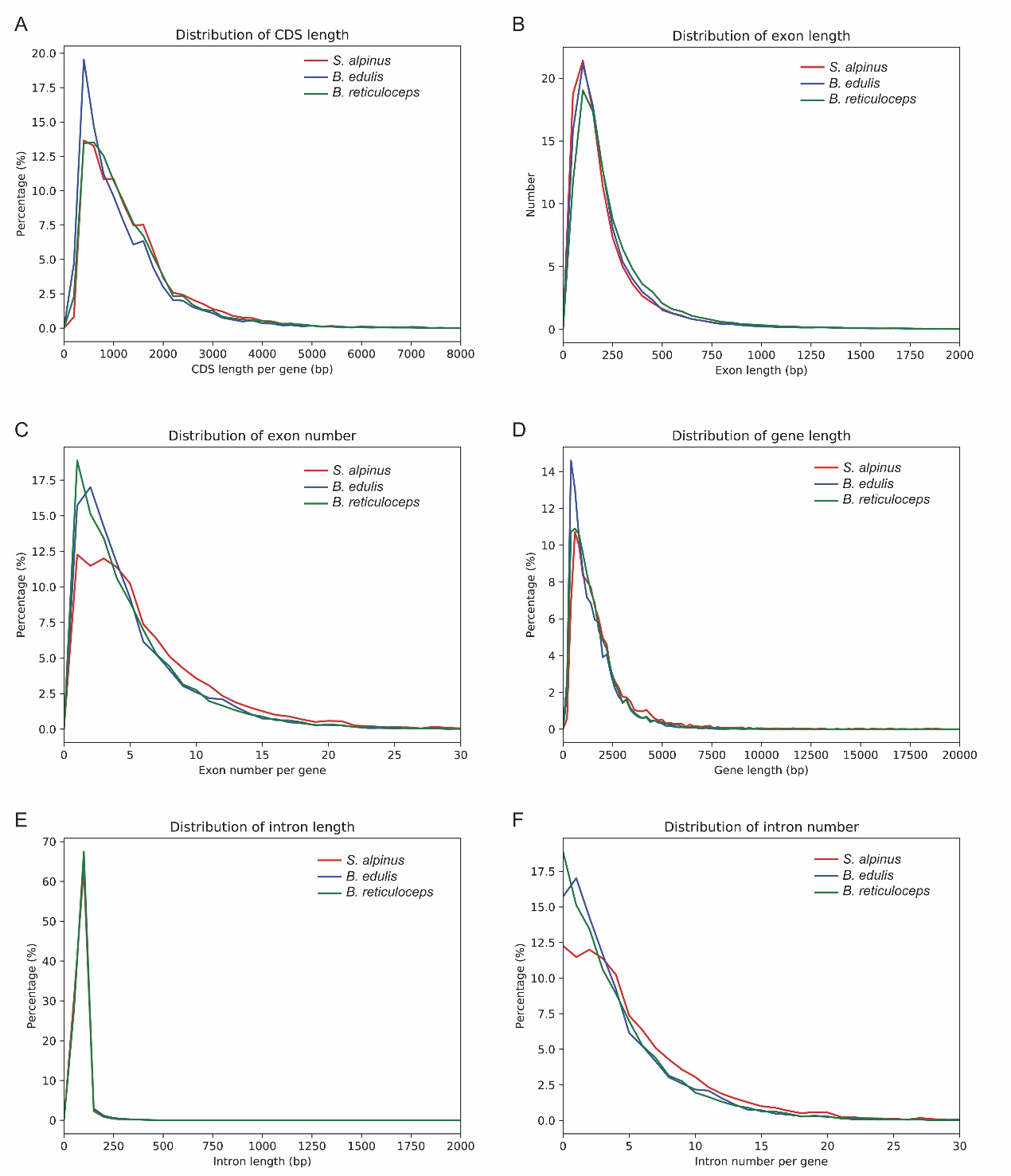


Figure S1. Characterization of distribution of CDS length (A), exon length (B), exon number (C), gene length (D), intron length (E), and intron number (F) in the genomeof *S alpinus* and two closely related species.

Figure S2. The annotation classification of NR corresponding of *S. alpinus* to the other species.


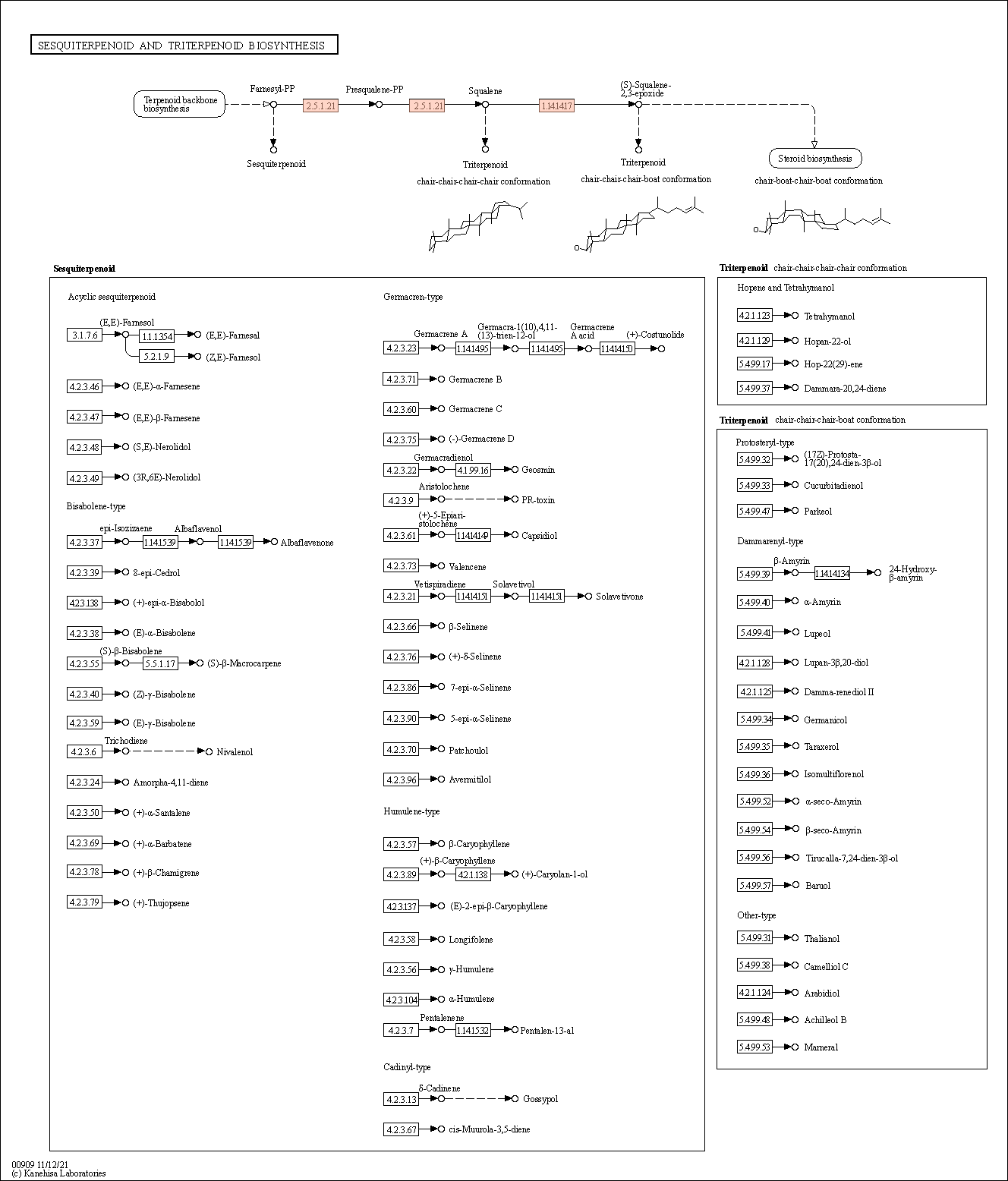


Figure S3. KEGG mapping of the sesquiterpenoid and triterpenoid biosynthesis pathways in *S. alpinus.*


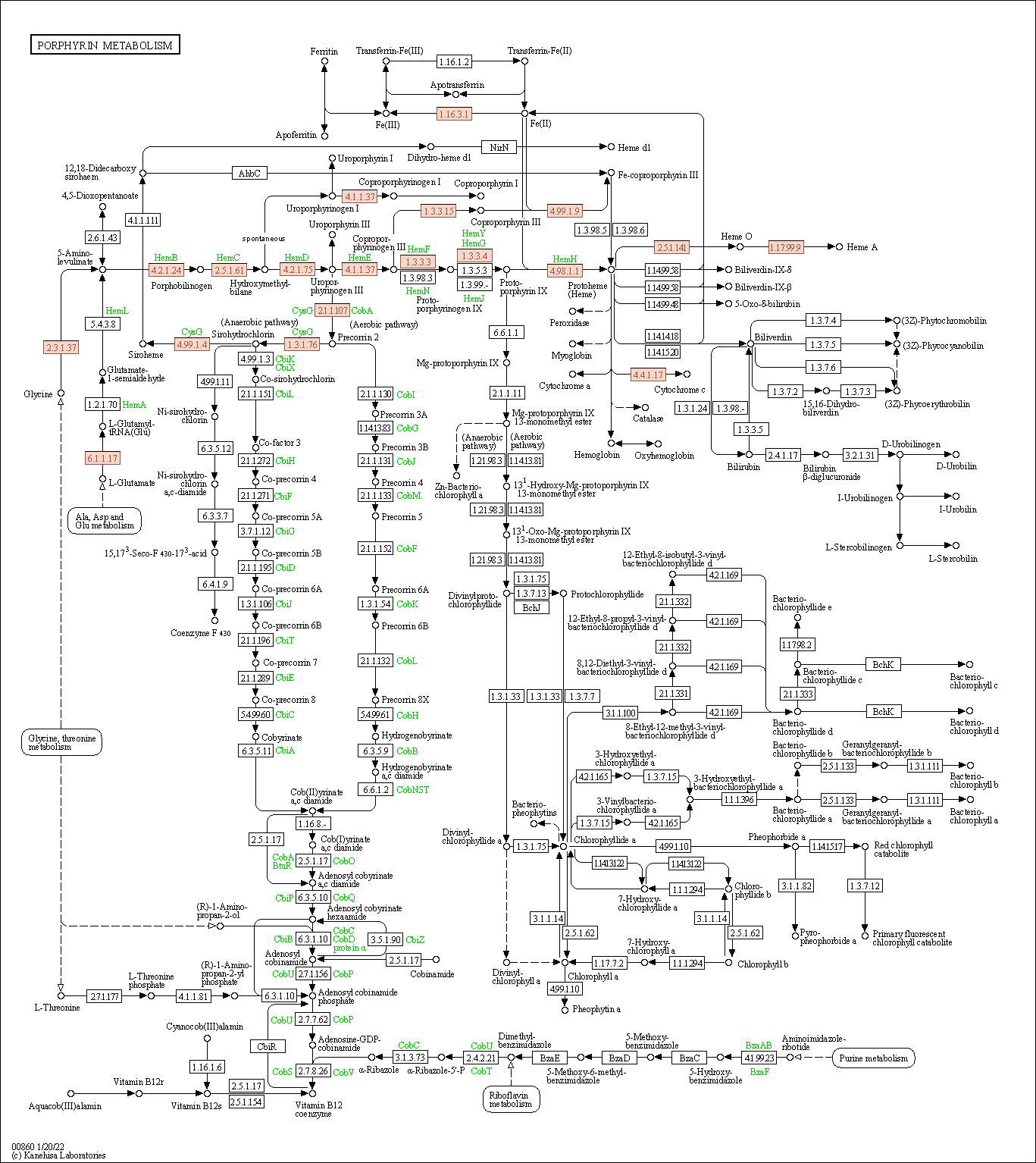


Figure S4. KEGG mapping of the porphyrin metabolism pathway in *S. alpinus.*


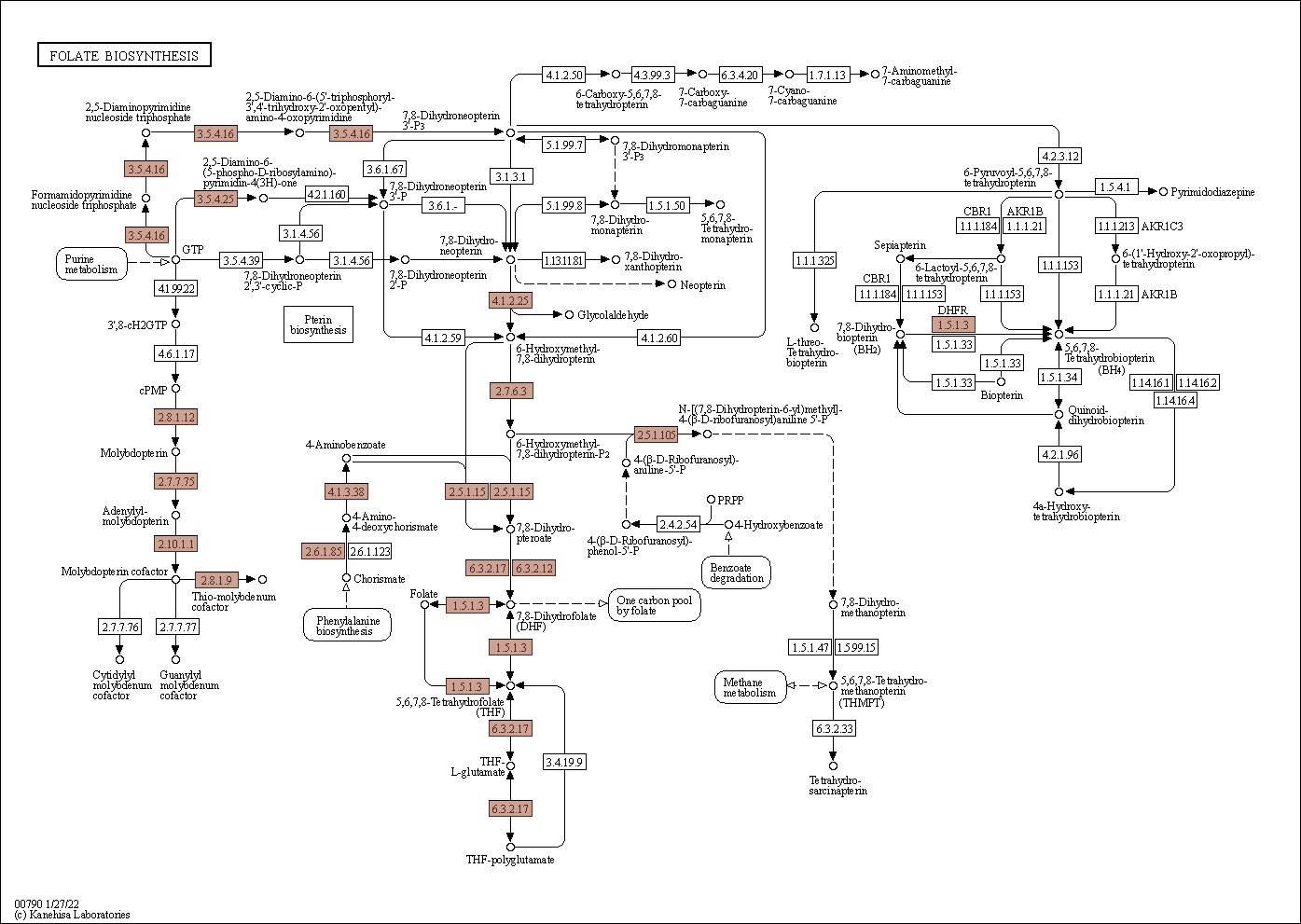


Figure S5. KEGG mapping of the folate biosynthesis pathway in *S. alpinus.*
